# Magnetic Resonance Imaging Profile of Blood–Brain Barrier Injury in Patients With Acute Intracerebral Hemorrhage

**DOI:** 10.1161/JAHA.113.000161

**Published:** 2013-06-21

**Authors:** Didem Aksoy, Roland Bammer, Michael Mlynash, Chitra Venkatasubramanian, Irina Eyngorn, Ryan W. Snider, Sandeep N. Gupta, Rashmi Narayana, Nancy Fischbein, Christine A. C. Wijman

**Affiliations:** 1 Stanford Neurocritical Care Program, Stanford Stroke Center, Stanford University Medical Center, Stanford, CA (D.A., M.M., C.V., I.E., R.W.S., R.N., C.C.W.); 2 Department of Radiology, Stanford University, Stanford, CA (R.B., N.F.); 3 Department of Neurology and Neurological Sciences, Stanford University, Stanford, CA (M.M., C.V., C.C.W.); 4 GE Global Research, Niskayuna, NY (S.N.G.)

**Keywords:** blood–brain barrier, dynamic contrast‐enhanced MRI, intracerebral hemorrhage, magnetic resonance imaging, stroke

## Abstract

**Background:**

Spontaneous intracerebral hemorrhage (ICH) is associated with blood–brain barrier (BBB) injury, which is a poorly understood factor in ICH pathogenesis, potentially contributing to edema formation and perihematomal tissue injury. We aimed to assess and quantify BBB permeability following human spontaneous ICH using dynamic contrast‐enhanced magnetic resonance imaging (DCE MRI). We also investigated whether hematoma size or location affected the amount of BBB leakage.

**Methods and Results:**

Twenty‐five prospectively enrolled patients from the Diagnostic Accuracy of MRI in Spontaneous intracerebral Hemorrhage (DASH) study were examined using DCE MRI at 1 week after symptom onset. Contrast agent dynamics in the brain tissue and general tracer kinetic modeling were used to estimate the forward leakage rate (K^trans^) in regions of interest (ROI) in and surrounding the hematoma and in contralateral mirror–image locations (control ROI). In all patients BBB permeability was significantly increased in the brain tissue immediately adjacent to the hematoma, that is, the hematoma rim, compared to the contralateral mirror ROI (*P*<0.0001). Large hematomas (>30 mL) had higher K^trans^ values than small hematomas (*P*<0.005). K^trans^ values of lobar hemorrhages were significantly higher than the K^trans^ values of deep hemorrhages (*P*<0.005), independent of hematoma volume. Higher K^trans^ values were associated with larger edema volumes.

**Conclusions:**

BBB leakage in the brain tissue immediately bordering the hematoma can be measured and quantified by DCE MRI in human ICH. BBB leakage at 1 week is greater in larger hematomas as well as in hematomas in lobar locations and is associated with larger edema volumes.

## Introduction

Spontaneous intracerebral hemorrhage (ICH) is defined as nontraumatic bleeding into the brain tissue. It is the deadliest and most disabling form of stroke and affects approximately a million people worldwide each year.^1–4^ Secondary brain injury and edema formation with resulting mass effect are thought to contribute to ICH‐related morbidity and mortality.^5–6^

The blood–brain barrier (BBB) is a neurovascular unit consisting of endothelial cells and astrocytic foot processes. “Tight junctions” between the endothelial cells regulate paracellular diffusion of water‐soluble substances between blood and brain compartments. In experimental models, the presence of intraparenchymal blood has been shown to increase BBB permeability and perihematomal edema formation via toxic effects of hemoglobin breakdown products.^7^ It has also been hypothesized that BBB disruption leads to angiogenesis around the hematoma, further contributing to the formation of vasogenic edema.^6^ Therefore, BBB injury may be an important pathophysiological factor in secondary brain injury caused by ICH and a potential target for therapeutic interventions.^8–9^

An intact BBB prevents larger molecules such as gadolinium diethylenetriamine penta‐acetic acid (Gd‐DTPA) from entering the brain tissue. Thus, leakage of gadolinium in the brain tissue reflects BBB disruption and can be used as a surrogate marker for BBB injury. In previous microvascular permeability studies of human brain tumors^10^ and tumor response to treatment,^11^ dynamic contrast‐enhanced MRI (DCE MRI) has been used to interrogate BBB permeability. DCE MRI has also been used to image BBB permeability in acute ischemic stroke patients and has been proposed as a potential method to identify patients with an increased risk for hemorrhagic transformation.^12^

In this study, we aimed to detect the presence of BBB injury and to quantify BBB leakage in acute spontaneous ICH in humans using DCE MRI. We also examined the relationships between the severity of BBB leakage and hematoma size, hematoma location, perihematomal edema volume, and underlying hemorrhage cause.

## Methods

### Patients and Magnetic Resonance Imaging

Twenty‐five prospectively enrolled patients who presented with a spontaneous ICH were included according to the following criteria: brain MRI was obtained in the first 2 days after symptom onset and again at 1 week after symptom onset; DCE MRI was included in the 1‐week MRI; no brain surgery was performed before the MRIs, and image quality was sufficient for analysis. Informed consent was obtained for study participation. The study was conducted in accordance with our institutional review board's requirements.

MR imaging was performed on a 1.5T GE Signa Excite scanner (GE Healthcare). Two time‐series of images were acquired using a spoiled gradient echo (SPGR) sequence for the baseline and follow‐up scans. The initial time‐series of images (ie, dynamic scan) used a low‐flip angle (5°) and had 5 time points. The second dynamic scan used a flip angle of 30° and had 30 time points acquired every 14 seconds, totaling to a scan time of 9 minutes. The latter scan was accompanied by the administration of 0.1 mmol/L per kg bodyweight Gd‐DPTA administered intravenously at an injection rate of 3 mL/s after the baseline scan. The 5° low‐flip angle time points with the 5 matching baseline scans (ie, prior to contrast arrival) from the DCE MRI scan with a flip angle of 30° served to map the native T1 times (needed for determining the contrast agent concentration) using a dual‐angle method.^13–14^ Slices at the edges of the three dimensional (3D) slab were discarded and those in the homogeneous center of the slab were used to avoid errors from flip angle variation or the slice profile across the 3D volume. With the exception of flip angle and number of dynamic scans, the scan parameters were identical for the low‐ and high‐flip angle and were as follows: TR/TE=7.8/3.4 ms, slice thickness=5 mm, number of slices used=12, field of view (FOV)=220 mm, rectangular FOV=70%, matrix size=192×256.

DCE MRI and BBB permeability measurements were performed on the second MRI performed at approximately 1 week after symptom onset. Hematoma volumes were measured with in‐house developed Interactive Data Language (IDL) software on the fluid attenuated inversion recovery (FLAIR) images of the 1 day MRI and dichotomized into small (<30 mL) and large (≥30 mL) similar to previous studies.^15–19^ Perihematomal edema volumes were measured at 1 day and 1 week using the FLAIR images. Scan parameters for the FLAIR sequence were TR/TE/TI=8800/100/2200 ms, slice thickness=5 mm, gap=0 mm, 22 slices, FOV=240 mm, matrix size=320×192, flip angle=90°.

### Postprocessing and MR Image Analysis

**Figure 1.**
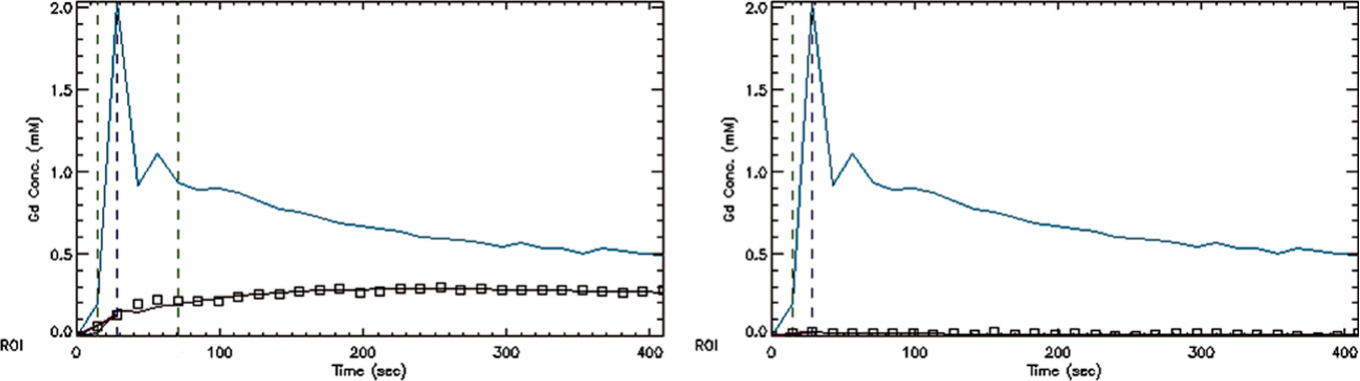
Gadolinium (Gd) concentration vs time graphs. Vascular input function (VIF) plot, data points (squares), and model fit for (A) representative lesion regions of interest (ROI) in the perihematomal rim and (B) mirror contralateral control ROI. Negligible (in the noise range) contrast material uptake can be seen in the ROI measurements in the control ROI in the hemisphere opposite from the hematoma.

DCE MR images were transferred to a computer workstation for postprocessing and analysis. First, motion‐correction of time‐series data and subsequent coregistration of the low‐ and high‐flip angle dynamic scans were performed using in‐house software developed in MATLAB (Mathworks). Second, hematoma ROIs were outlined on the FLAIR images of the day‐1 MRI. Perihematomal edema ROIs were outlined on the FLAIR images of the day‐1 and week‐1 MRI. Volumes were calculated using an in‐house developed IDL‐based software (ITT Visual Information Solutions). Third, acquired DCE MRI data were analyzed using IDL based investigational pharmacokinetic analysis software (KinMod CINEtool, GE Healthcare). Signal intensity over time curves were generated for perihematomal and contralateral mirror control ROIs. In the perihematomal ROIs, the contrast agent was expected to accumulate over time as a surrogate marker of BBB leakage, whereas contrast uptake was not expected to occur within the mirror control ROIs in the opposite hemisphere (Figure 1).

**Figure 2.**
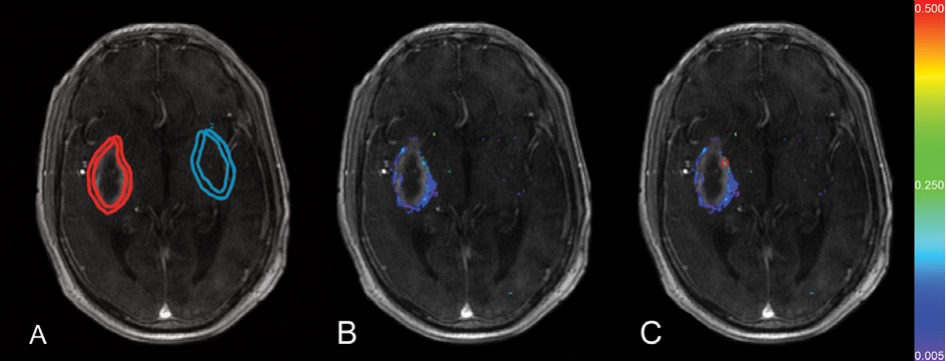
A, Lesion regions of interest (ROI) (red) surrounding the hematoma and control ROI (blue) on the contralateral side. B, Blood–brain barrier (BBB) permeability map (C) “hot spot” ROI (red) outlined on permeability map. Color coded K^trans^ values are within the range of 0.005 to 0.500 min^−1^, purple being 0.005 min^−1^, blue 0.075 min^−1^, light blue 0.150 min^−1^, green 0.250 min^−1^, light green 0.325 min^−1^, yellow 0.400 min^−1^, and red 0.500 min^−1^.

Details of the pharmacokinetic analysis are as follows: First, a “lesion ROI” was selected to cover the entire perihematomal rim, ≈3 mm wide, surrounding the hematoma on 3D SPGR T1 weighted DCE MR images. On this sequence at 1 week after symptom onset, the center of the hematoma is typically isointense due to intracellular deoxyhemoglobin and the periphery of the hematoma is hyperintense due to intracellular methemoglobin. Within the thicker and more uniform hyperintense perihematomal region, a thinner rim with variable permeability increase is observed. Using this variability in permeability, the leakiest section of the rim was outlined on the K^trans^ maps to generate a second, so called “hot spot,” ROI (Figure 2). Third, for both the “lesion ROI” and the “hot spot” ROI, corresponding “control ROIs” were placed on the homologous contralateral (mirror) locations.

The KinMod software utilizes a 2‐compartment general kinetic model to represent the Gd‐DTPA dynamics measured in the tissue. These 2 compartments are the vascular space (VS) and the extracellular extravascular space (EES). Fractions of these 2 compartments are represented in each voxel of the MR image.^20–21^ Changes in the tissue tracer concentration can be calculated by determining the difference between the leakage of VS and EES, and the transfer of tracer material from EES to VS using the following modeling approach^20–21^:



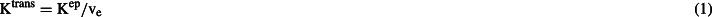
1




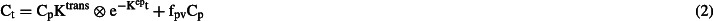
2


Here, C_t_ is the tissue tracer concentration, C_p_ the tracer concentration in the blood plasma (vascular input function), K^trans^ the forward leakage rate (permeability), K^ep^ the transfer constant from the EES to the vascular space, f_pv_ the fractional plasma volume, and v_e_ the volume of the EES per unit tissue volume.

By means of a vascular input function (VIF), obtained from a large feeding artery, one is able to directly use the change of gadolinium concentration over time and fit the model to the data obtained.^20^ Estimates of model parameters, including the forward leakage rate (K^trans^), which represents the BBB permeability, were obtained for the lesion and control ROIs using the analysis software. Permeability maps were generated using the derived forward leakage parameters from the general kinetic model.^21^

### Statistical Analysis

Values reported are in mean (±SD), median (interquartile range) and proportions. Estimated K^trans^ values for the lesion ROIs adjacent to the hematoma and the mirror control ROIs on the contralateral side were analyzed using Wilcoxon signed rank test. Mann–Whitney U test was used to compare the K^trans^ values for small and large hemorrhages and to compare the K^trans^ values of hemorrhages in cerebellar, lobar, and deep locations. Regression analysis with weighted least squares was used to compare the K^trans^ values of hematomas with different presumed causes. Tests were 2‐tailed and the significance level was chosen as α ≤0.05. Statistical analyses were performed using MATLAB Statistics Toolbox (Mathworks) and IBM SPSS 19.0 (IBM).

## Results

Of 30 prospectively enrolled ICH patients, 5 with motion‐degraded MRIs were excluded. Age of the 25 included patients was 64.8 (±13.8) years, 64% were female, median (Q1 to Q3) admission National Institutes of Health stroke scale score (NIHSS) was 14 (6 to 19), and admission Glasgow coma scale score (GCS) was 10 (7 to 14) (Table 1). The presumed cause of ICH was hypertension in 10 (40%), cerebral amyloid angiopathy (CAA) in 5 (20%), vascular malformation in 3 (12%), illicit drug use in 2 (8%), posterior reversible encephalopathy syndrome in 2 (8%), vasculitis in 1 (4%), brain tumor in 1 (4%), and hemorrhagic transformation of an ischemic stroke in 1 (4%). Twelve patients had a lobar hematoma, 8 had deep hematomas, 1 had a lobar and deep hematoma, and 4 had cerebellar hemorrhages. Median ICH volume was 34.5 mL (12.7 to 65.5). BBB leakage was measured at 7.9 (±1.4) days after symptom onset.

**Table 1.** Demographics (n=25)

Age, mean±SD (y)	64.8±13.8
Female, n (%)	16 (64)
Admission NIHSS, median (Q1 to Q3)	14.0 (6.0 to 19.0)
Admission GCS, median (Q1 to Q3)	10.0 (7.0 to 14.3)
History of HTN, n (%)	22 (88)
Diabetes, n (%)	4 (16)
History of alcohol abuse, n (%)	4 (16)
History of stroke, n (%)	5 (20)
Antithrombotic treatment	
ASA, n (%)	4 (16)
Warfarin, n (%)	1 (4)
Plavix, n (%)	1 (4)
Hematoma volume (mL), median (Q1 to Q3)	34.5 (12.7 to 65.5)

NIHSS indicates National Institutes of Health Stroke Scale; GCS, Glasgow Coma Scale; HTN, hypertension; ASA, acetylsalicylic acid.

**Figure 3.**
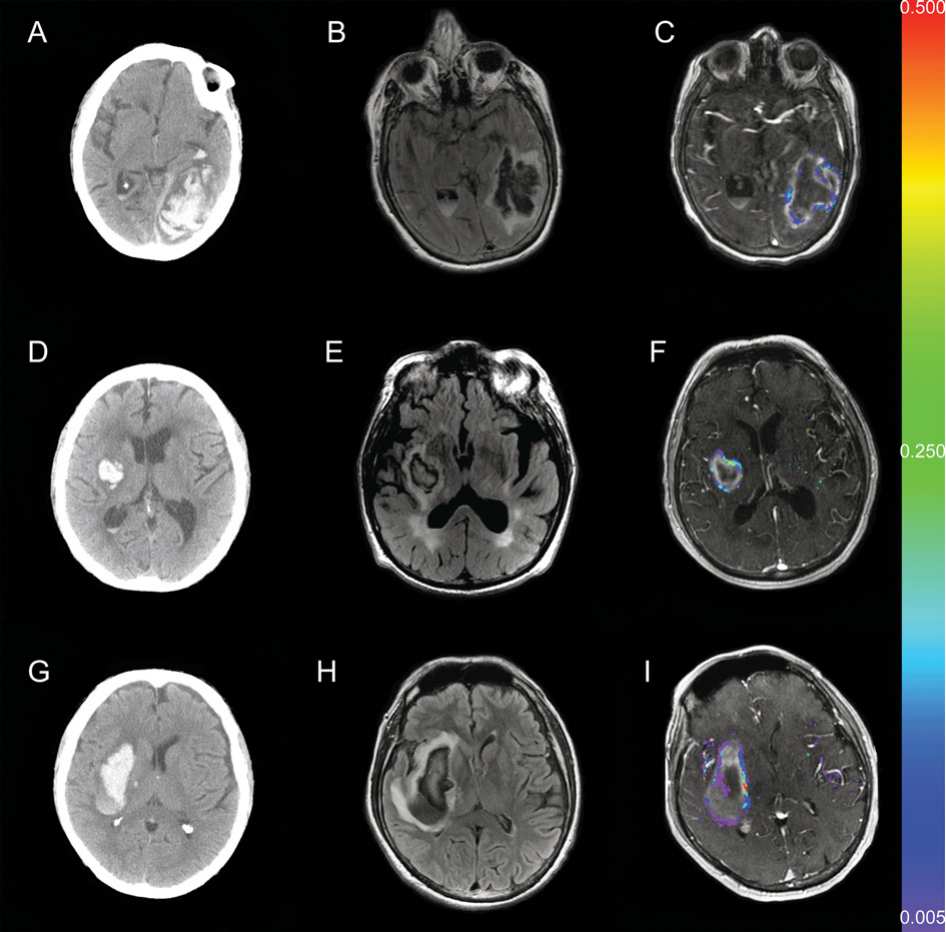
Admission brain CT, 1‐day FLAIR MRI and 1 week color coded permeability maps of a patient with a lobar hematoma (A, B, C), a patient with a small basal ganglionic (deep) hematoma (D, E, F), and a patient with a large basal ganglionic (deep) hematoma (G, H, I). BBB disruption around the hematoma at 1 week after symptom onset is readily visible on the permeability maps in all 3 patients. Color coded K^trans^ values are as described in the legend of Figure 2. CT indicates computed tomography; FLAIR, fluid attenuated inversion recovery; MRI, magnetic resonance imaging; BBB, blood–brain barrier.

BBB permeability maps were constructed and measurements were performed on a total of 163 slices covering the hematomas of the 25 patients. Increased BBB permeability was observed in the rim of brain tissue immediately adjacent and surrounding the hematoma in all patients. Contrast agent uptake was not observed in the hematoma itself. Areas of increased permeability surrounding the hematoma were identifiable on the color‐coded K^trans^ maps (Figures 2 and 3). Representative permeability maps demonstrating increased K^trans^ are shown in Figure 3C, 3F, and 3I. Corresponding slices of the admission computed tomography images and the FLAIR images acquired at 1.5 (±1.1) days after symptom onset are also included to show hematoma volume and location, and the amount of perihematomal vasogenic edema (Figure 3).

As mentioned earlier, all patients had increased BBB permeability in the rim of brain tissue directly adjacent to the ICH without leakage in the hematoma itself. Furthermore, 1 patient with hemorrhagic transformation (HT) of an ischemic stroke had evidence of BBB leakage throughout the entire area of infarction rather than in the perihematomal rim alone. This pattern of increased permeability in subacute ischemic infarcts is due to BBB injury in the ischemic stroke core and consistent with findings of permeability increases found in previous HT studies.^12,22^

Comparison of the model parameter K^trans^ revealed a significant difference between the lesion and control mirror ROIs in all analyses (*P*<0.0001) (Table 2). Permeability of the region surrounding the hematoma was calculated for each patient. The median (Q1 to Q3) K^trans^ for the lesion ROI was 0.055 min^−1^ (0.020 to 0.101) and for the control mirror ROI 0.000 min^−1^ (0 to 0.014) (*P*<0.0001). Thirteen of the 25 (52%) patients did not have any BBB leakage (K^trans^=0 min^−1^) in their control mirror ROI; the other 12 patients had an infinitesimal increase in BBB permeability in their control mirror ROI on one or more images.

**Table 2.** BBB Permeability Measured in the Entire Rim Surrounding the Hematoma and the Leakiest Section of the Rim and the Corresponding Contralateral Brain Regions (Controls)

	K^trans^ in Lesion ROI (min^−1^)	K^trans^ in Control ROI (min^−1^)	*P* Value
Entire rim	0.055 (0.020 to 0.101)	0.000 (0 to 0.014)	<0.0001
Leakiest region	0.162 (0.101 to 0.316)	0.003 (0 to 0.014)	<0.0001

Values reported are medians (Q1 to Q3), n=25. BBB indicates blood–brain barrier; ROI, regions of interest.

### BBB Permeability Versus Hematoma Volume

**Figure 4.**
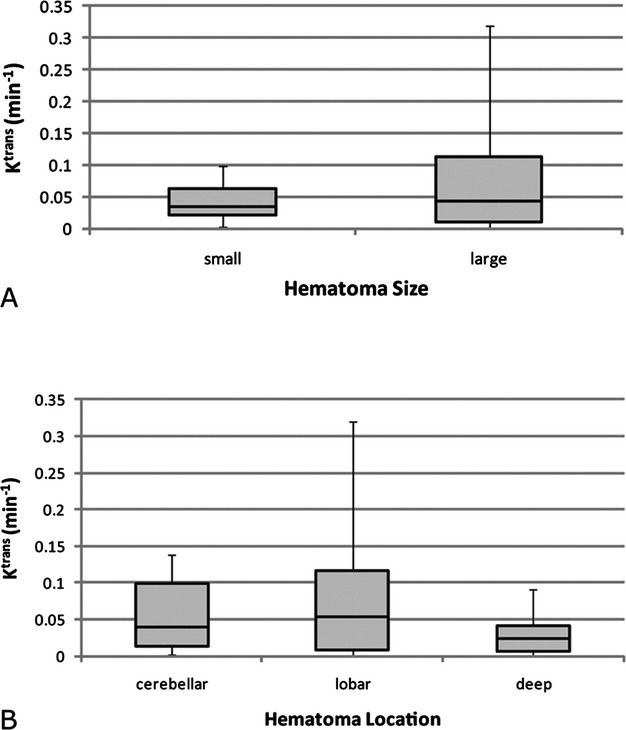
K^trans^ values including all magnetic resonance imaging (MRI) slices through the hematoma, by hematoma size and location. A, Large hematomas (≥30 mL) had higher blood–brain barrier (BBB) permeability surrounding the hematoma (*P*<0.005), and a wider range than small hemorrhages. B, BBB permeability around lobar hemorrhages was higher than permeability around deep hemorrhages (*P*<0.005). BBB permeability around cerebellar hemorrhages tended to be more similar to the lobar hemorrhages (*P*=0.57) than the deep hemorrhages (*P*=0.09). In the box plot, central horizontal lines are the medians, edges of the boxes are Q1 and Q3, whiskers extend to minimum and maximum values.

Of the 25 patients, 12 had large (≥30 mL) hemorrhages (69±27 mL) and 11 had small (<30 mL) hemorrhages (14±9 mL); 2 patients were excluded from this analysis: 1 did not have a day one scan, and 1 had a large intraventricular hemorrhage (IVH) with small intraparenchymal component. K^trans^ in the hematoma rim was 0.028 min^−1^ (0 to 0.057) for the small (<30 mL) hematomas and 0.051 min^−1^ (0.012 to 0.121) for the large (≥30 mL) hematomas (*P*<0.005). Thus, the larger hematomas had higher BBB permeability and also had a wider range in their BBB permeability values compared to the smaller hematomas (Figure 4A).

### BBB Permeability Versus Hematoma Location

Twelve patients had lobar hemorrhages (n=87 slices), 7 had deep hemorrhages (n=45 slices) and 4 had cerebellar hemorrhages (n=18 slices). Two of the 25 patients were excluded from this analysis: 1 with such a large hemorrhage that it could not be categorized in a single location category and 1 with a large intraventricular hemorrhage (IVH) with a small intraparenchymal component.

BBB permeability in lobar hemorrhages was higher than in deep hemorrhages regardless of similar hematoma volumes, 38 mL (18 to 74) and 42 mL (12 to 70), respectively (*P*=0.93). K^trans^ around the hemorrhage in lobar regions was 0.053 min^−1^ (0.008 to 0.116) whereas K^trans^ surrounding deep hemorrhages was 0.023 min^−1^ (0.006 to 0.042) (*P*<0.005). Furthermore, the BBB permeability at the leakiest section of the rim was also higher in lobar hematomas at 0.208 min^−1^ (0.051 to 0.508) than in deep hematomas at 0.080 min^−1^ (0.039 to 0.176) (*P*<0.01). Lobar hemorrhages also had more variance in their BBB permeability compared to cerebellar and deep hemorrhages, 0.025 versus 0.003 and 0.002, respectively.

K^trans^ values around the cerebellar hematomas were 0.04 min^−1^ (0.013 to 0.099) and at the leakiest section of their rim 0.166 min^−1^ (0.079 to 0.323). Although no significant difference was found in BBB permeability between cerebellar and lobar hemorrhages (*P*=0.57), and between cerebellar and deep hemorrhages (*P*=0.09), BBB leakage around cerebellar hemorrhages tended to be more similar to lobar than to deep hemorrhages (Figure 4B).

### BBB Permeability and ICH Cause

BBB permeability around hemorrhages presumably caused by chronic hypertension, 0.015 min^−1^ (0 to 0.061), was lower than around hematomas attributed to CAA or other nonhypertensive causes, 0.054 min^−1^ (0.027 to 0.097) (*P*<0.001). When corrected for hematoma volume using regression analysis with weighted least squares, ICH cause had a strong trend predicting permeability (*P*=0.068).

### BBB Permeability Versus Edema Volume

**Figure 5.**
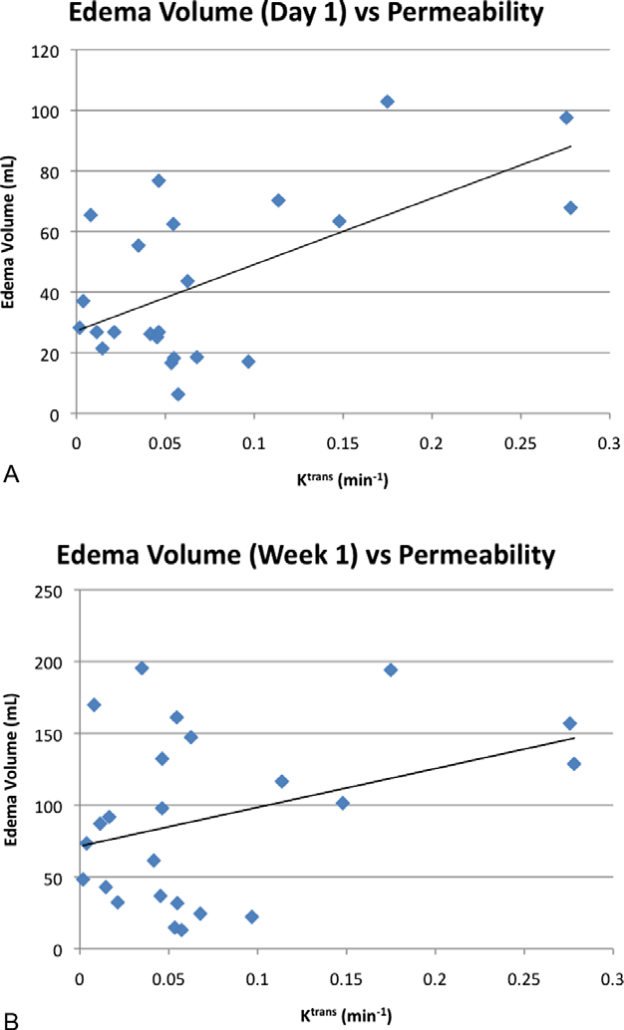
Edema volume vs blood–brain barrier (BBB) permeability. Mean K^trans^ and edema volume are shown for each patient. Higher K^trans^ is associated with larger edema volumes (A) on day 1 (ρ=0.62) and (B) at 1 week after intracerebral hemorrhage (ICH) onset (ρ=0.35).

BBB permeability and its relationship with edema volume at day‐1 and edema volume at 1 week were studied in 23 and 24 patients, respectively. One patient who did not have a day‐1 scan, and 1 patient with a large IVH and a small intraparenchymal component were excluded from this analysis. Higher BBB permeability was associated with larger edema volumes both at day‐1 and at 1 week. The correlation was stronger at 1 day than at 1 week (ρ=0.62 and 0.35, *P*=0.002 and 0.09, respectively) (Figure 5).

## Discussion

Using DCE MRI we demonstrated the presence of BBB leakage in a prospective cohort of 25 spontaneous ICH patients and quantified its severity at 1 week after ICH onset. This is a unique report as BBB leakage is challenging to study in vivo in human ICH and published data in this area of study are scarce. We found considerable BBB leakage in the rim of brain tissue immediately adjacent and surrounding the hematoma, at 8 days after ICH onset, but typically not in the hematoma itself, and not in the contralateral mirror brain region. A recent study of human ICH reported perihematomal contrast enhancement in 60% of ICH patients at 5 days after symptom onset.^23^

One potential explanation for the absence of contrast leakage in the hematoma itself is that the blood clot prevents contrast agent uptake. In a rat model of ICH, Knight et al^24^ reported increased BBB permeability in the hematoma core and the rim at 7 days after ICH onset. It needs to be kept in mind however that animal models of ICH, such as direct infusion of autologous blood in the absence of vessel rupture, do imperfectly reflect human pathophysiology. An infinitesimal increase in BBB permeability in the contralateral mirror ROI in the opposite hemisphere was observed in half of our patients. This increase was limited to a few specks randomly distributed and entirely different from the sizable BBB leakage surrounding the hematoma (Figure 3). This observation may be artifactual due to image noise and the presence of blood vessels carrying gadolinium near the control ROI. Alternatively, the presence of ICH may cause a true subtle increase of BBB permeability in brain regions remote from the hematoma in a subset of patients.

Our analysis revealed a significant relationship between BBB leakage and hematoma size. Large hematomas (≥30 mL) had more BBB leakage around the hematoma and an increased variance in BBB permeability severity than small hematomas. This finding seems plausible because larger hematomas contain greater amounts of blood products causing more BBB injury than small hematomas. This observation is also congruent with the observation that larger hematomas produce greater absolute edema volumes than small hemorrhages.^25^ Blood–brain barrier leakage differed by hemorrhage location, with lobar hemorrhages being associated with more BBB permeability and higher variability in BBB leakage than deep hemorrhages, irrespective of hematoma size. The difference in BBB leakage may be caused by differences in vascularity and local tissue pressures in different brain regions. Alternatively, it is possible that different causes of ICH affect BBB permeability in different ways. Since lobar hemorrhages in our study were more often attributed to CAA and deep hematomas to hypertension, we could not determine whether the observed differences in BBB permeability were caused by differences in ICH pathophysiology or ICH location. This area needs further study. Understanding the association between hematoma location, hematoma cause, and degree of BBB permeability will help increase our understanding of the mechanisms causing BBB injury and other secondary brain injuries in ICH.

Reports on the timing of peak perihematomal edema volume in human ICH vary considerably. Some authors report that peak edema volume occurs within the first few days,^26^ but others in the first week^19^ or the second and third weeks.^25,27–28^ We chose to measure BBB leakage at 1 week after symptom onset, because in a previous study of ICH patients with serial MR imaging we found that edema volume measured on FLAIR MRI peaks right after the first week.^25^ At this time point not only is the perihematomal edema typically present but BBB leakage is also likely to be observed. Furthermore, from a pragmatic point of view 1 week is a good time point to consistently be able to image a prospective ICH patient cohort using MRI.

Although we found a correlation between higher BBB permeability and larger edema volumes, this relationship was fair at best. This finding suggests that BBB permeability is one, but not the only variable, associated with perihematomal edema formation and that other factors are likely to be important. There is some evidence to suggest that hematological factors play a role in the amount of edema formation.^25^

A limitation of this study is that we performed a single measurement of BBB permeability rather than serial measurements. Animal studies suggest that an increase in BBB permeability begins within hours of ICH onset and continues to increase up to 48^26^ and 72 hours.^29^ In some of these models the amount of BBB leakage starts to decrease gradually after an initial peak in the first few days, but continues to be observed up to 14 days.^24^ In one model, BBB leakage at 1 week after ICH onset was measured to be similar to BBB leakage at 1 day.^29^ Given the paucity of data on BBB permeability in human ICH, serial measurements would have been ideal; however, it is very challenging to obtain these data in humans due to the length of the MRI sequences (≈9 minutes). This is especially true for MRI scans obtained in the first day or 2 after ICH onset when patients are often agitated and have a hard time staying still. Even though motion correction was done for all MR images, 5 patients (17% of our patient population) had to be excluded from this study due to inferior image quality caused by motion artifact. Furthermore, it is not uncommon for patients' condition to not allow MR imaging to be done on a predetermined schedule, especially earlier in the course of ICH. Although it is very challenging to gather these data, sequential dynamic imaging of ICH patients at multiple time points is likely to shed additional light on the evolution of BBB disruption and secondary injury following spontaneous ICH in humans and deserves further study.

Other limitations of this study are the small sample size and potential inaccuracies in estimating permeability introduced by paramagnetic blood products from the hematoma. Multiple slices covering the hemorrhage were acquired for each patient. Although multiple slices significantly increases the number of data points, our sample size does not allow for an assessment of the correlation of severity of BBB leakage and clinical worsening or outcome, which deserves further study. Furthermore, although BBB leakage was higher in large and lobar hemorrhages, we measured BBB leakage in a predefined hematoma rim; therefore, we cannot comment on potential subtle differences in total perihematomal tissue volume with BBB compromise by hematoma size or location; however, from visual inspection of our permeability maps it is evident that, at 1 week after ICH onset, substantially increased BBB permeability is limited to approximately a 1 to 3 mm rim immediately adjacent to the hematoma. Finally, our study aimed at imaging and quantifying the severity of BBB injury in a consecutive series of patients with spontaneous (ie, nontraumatic) ICH. It should be noted that our cohort was heterogeneous in terms of the presumed cause of ICH and did not only include patients with primary ICH (ie, ICH caused by hypertension or CAA).

In summary, in this study we found evidence of BBB leakage following ICH in humans using DCE MRI. Increased BBB permeability was observed at the rim of tissue immediately surrounding the hematoma at 1 week after symptom onset in all patients. Large hematomas produced more severe and more variable BBB leakage than small hematomas. Furthermore, BBB leakage around lobar hemorrhages was higher than around deep hemorrhages independent of hematoma volume. Higher BBB leakage was associated with larger edema volumes. Quantification of BBB injury following human ICH may reflect the severity of secondary brain injury caused by ICH and holds promise as a surrogate endpoint in future trials testing interventions aimed at limiting perihematomal tissue injury.
